# Aberrant Functional Connectivity Architecture in Participants with Chronic Insomnia Disorder Accompanying Cognitive Dysfunction: A Whole-Brain, Data-Driven Analysis

**DOI:** 10.3389/fnins.2017.00259

**Published:** 2017-05-11

**Authors:** Ran Pang, Yafeng Zhan, Yunling Zhang, Rongjuan Guo, Jialin Wang, Xiao Guo, Yong Liu, Zhiqun Wang, Kuncheng Li

**Affiliations:** ^1^Department of Radiology, Xuanwu Hospital, Capital Medical UniversityBeijing, China; ^2^Department of Radiology, Dongfang Hospital, Beijing University of Chinese MedicineBeijing, China; ^3^Brainnetome Center, Institute of Automation, Chinese Academy of SciencesBeijing, China; ^4^School of Biomedical Engineering, Southern Medical UniversityGuangzhou, China; ^5^Department of Neurology, Dongfang Hospital, Beijing University of Chinese MedicineBeijing, China; ^6^School of Clinical Medicine, Beijing University of Chinese MedicineBeijing, China; ^7^National Laboratory of Pattern Recognition, Institute of Automation, Chinese Academy of SciencesBeijing, China; ^8^Center for Excellence in Brain Science and Intelligence Technology, Institute of Automation, Chinese Academy of SciencesBeijing, China; ^9^University of Chinese Academy of SciencesBeijing, China; ^10^Beijing Key Laboratory of Magnetic Resonance Imaging and Brain InformaticsBeijing, China

**Keywords:** chronic insomnia disorder, cognitive impairment, functional connectivity, resting-state fMRI

## Abstract

**Objectives:** Although it is widely observed that chronic insomnia disorder (CID) is associated with cognitive impairment, the neurobiological mechanisms underlying this remain unclear. Prior neuroimaging studies have confirmed that a close correlation exists between functional connectivity and cognitive impairment. Based on this observation, in this study we used resting-state functional magnetic resonance imaging (rs-fMRI) to study the relationship between whole brain functional connectivity and cognitive function in CID.

**Methods:** We included 39 patients with CID and 28 age-, gender-, and education-matched healthy controls (HC). Abnormalities in functional connectivity were identified by comparing the correlation coefficients for each pair of 116 brain regions between CID and HC.

**Results:** Cognitive impairment was associated with reduced subjective insomnia scores after controlling for age, gender, and educational effects. Compared with HC, patients with CID had larger negative correlations within the task-negative network [medial prefrontal cortex (mPFC), precuneus, inferior temporal gyrus, cerebellum, and superior parietal gyrus], and between two intrinsic anti-correlation networks (mPFC and middle temporal gyrus; supplementary motor area and cerebellum). Patients with CID also had decreased positive correlations within the default mode network (DMN), and between the cerebellum and DMN, which mainly comprises the mPFC and posterior cingulated cortex. There were positive correlations of decreased positive connectivity with subjective sleep scores and MMSE scores, and increased negative correlations between the task-negative-network and MMSE scores in CID.

**Conclusions:** Using rs-fMRI, our results support previous observations of cortical disconnection in CID in the prefrontal and DMN networks. Moreover, abnormal correlations within the task-negative network, and between two intrinsically anti-correlation networks, might be important neurobiological indicators of CID and associated cognitive impairment.

## Introduction

Epidemiological studies have reported that about 9.2% of adults in China suffer from insomnia or sleep disorder (Xiang et al., 2008; Chen et al., 2015). Chronic insomnia disorder (CID), a common sleep-wake disorder, is associated with cognitive impairment (Fortier-Brochu et al., 2012; Muto et al., 2012; Potvin et al., 2012; Lim et al., 2013; Mander et al., 2014; Yaffe et al., 2014). It has been observed that fewer hours of sleep, as well as lower sleep quality, are associated with increased β-amyloid deposits in the brains of older people (Ju et al., 2013; Spira et al., 2013; Ooms et al., 2014). In addition, it has been reported that aging sleep disorder is associated with accelerated β-amyloid deposits in the prefrontal cortex, which is related to impaired memory consolidation during the night (Lucey and Holtzman, 2015). Therefore, studying the pathophysiological mechanisms of CID and its influence on cognitive function has attracted researchers' interest.

Based on the ascending reticular structure and cortical hyperarousal theory, previous work has confirmed that the prefrontal lobe and hippocampus show relatively reduced metabolism from waking to a non-REM sleep state in patients with insomnia than that in healthy people, while cortical and subcortical regions have decreased metabolism in the arousal state (Nofzinger et al., 2004; Altena et al., 2008; Riemann et al., 2010; Morin and Benca, 2012). The abnormal metabolism of prefrontal and hippocampal regions in insomnia may cause cognitive dysfunction (Mosconi et al., 2005, 2008; Li et al., 2008).

Some earlier morphological studies using magnetic resonance imaging (MRI) observed a reduced hippocampal volume in patients with insomnia compared with healthy participants (Riemann et al., 2007). However, this result is contentious because other studies have not replicated evidence for hippocampal volume loss in insomnia (Winkelman et al., 2010; Spiegelhalder et al., 2013). Subsequently, some studies reported reduced gray matter density and volume in the frontal lobes in insomnia (Altena et al., 2010; Joo et al., 2013; Mander et al., 2013). This is consistent with observed performance deficits in executive function (Joo et al., 2013). Furthermore, a longitudinal clinical trial with a larger sample demonstrated that insomnia leads to an increased cortical atrophy rate within widespread prefrontal, temporal, and parietal regions (Sexton et al., 2014). Atrophy within these cognition-related regions implies that poor sleep quality may be related to multifactorial cognitive deficits.

A study combining MRI and electroencephalography (EEG) reported that prefrontal atrophy is associated with reduced non-rapid eye movement (NREM) and slow wave activity (SWA), and sustained activation of the hippocampus (rather than progressive hippocampus-cortex memory conversion; Mander et al., 2013). This indicates that cognitive decline is associated with sleep disorder derived from the aging brain (structure atrophy). Moreover, neuroimaging studies have shown that the loss of brain functional connectivity is an important cause of cognitive impairment in patients with Alzheimer's disease (AD; Wang et al., 2007; Zhou et al., 2008; Binnewijzend et al., 2012; Serra et al., 2016). Therefore, identifying changes in brain connectivity might help to elucidate the mechanisms underlying CID and its associated cognitive impairment.

Using resting-state functional MRI (rs-fMRI), studies have identified the existence of functional disconnection within the prefrontal lobe and default mode network (DMN), and between the DMN and its negative-correlation network in sleep deprivation samples (De Havas et al., 2012; Verweij et al., 2014). In addition, a longitudinal study integrating structural and functional MRI reported cortical atrophy in the anterior cingulate gyrus, precentral gyrus, and anterior lateral prefrontal cortex, with structural disconnection between anterior and posterior regions of the DMN in patients with CID compared with healthy subjects (Suh et al., 2016).

Work focusing on the prefrontal lobe or DMN using predefined seed regions has indicated that there is regional brain dysfunction or special network disruption in insomnia. However, no study has investigated whole brain resting-state functional connectivity changes, which could avoid the limitations of using a seeded region of interest method. Furthermore, whole brain connectivity could reveal more comprehensive brain network changes and improve our understanding of the pathophysiology of brain disease (van de Ven et al., 2004; Fox and Raichle, 2007; Liu et al., 2008a,b). Considering the possibility that abnormalities in functional connectivity may exist in widely distributed brain regions in CID, it is helpful to study functional connectivity from the perspective of the whole brain to elucidate the mechanisms underlying CID and its associated cognitive impairment.

We hypothesize that, compared with age-matched cognitively healthy participants, there are measurable differences in functional connectivity between specific brain regions in patients with insomnia, and that these differences are associated with cognitive impairment. To verify this hypothesis, we investigated altered brain functional connectivity throughout the whole brain in 39 patients with CID and 28 age- and gender-matched healthy controls (HC). First, we examined functional connectivity within a well-defined automated anatomical labeling (AAL) template (Tzourio-Mazoyer et al., 2002). Second, we evaluated differences in the correlation coefficients for pairs of brain regions between CID and HC to identify altered connectivity. We also aimed to investigate whether identified altered functional connectivity is affected by variation in clinical variables.

## Materials and methods

### Participants

Participants comprised patients at a neurology clinic and volunteers from Dongfang Hospital, Beijing, China. First, all participants underwent a clinical interview by an experienced CID neurologist (J.W.). They then underwent a laboratory blood test and neuropsychological examination. Patients and HC were matched for age, gender, and education level. Participants were strictly screened and signed consent forms before participation. The study protocol was approved by the ethics committee of Dongfang Hospital (JDF-IRB-2014035302).

Patients with CID underwent a complete physical and neurological examination, standard laboratory tests, and an extensive battery of neuropsychological assessments. These neuropsychological assessments included the Pittsburgh Sleep Quality Index (PSQI), Insomnia Severity Index (ISI), Hamilton Anxiety Scale (HAMA), Hamilton Depression Scale (HAMD), Mini-Mental State Examination (MMSE), Montreal Cognitive Assessment (MoCA), and Clinical Dementia Rating (CDR). They also underwent polysomnography (PSG). Note that the diagnosis of CID met the criteria of the fifth edition of the Diagnostic and Statistical Manual of Mental Disorders (DSM-5) and the third edition of the International Classification of Sleep Disorders (ICSD-3; Edinger et al., 2004). To discount the potential side effects of oral medication, all patients with CID had not received treatment within the previous 3 months.

All patients with CID met the following criteria: (1) history of chronic insomnia of at least 3 months; (2) sleeps <7 h a day; (3) PSQI score ≥5 and ISI score ≥14; (4) able to cooperate with cognitive testing; (5) no clinical history of stroke or other severe cerebrovascular disease; and (6) no more than one lacunar focus, without patchy or diffuse leukoaraiosis, on neuroradiological assessment of conventional MR images.

Healthy controls underwent a routine physical examination to discount physical and basic metabolic disease. They then completed a series of neuropsychological assessments (MMSE, MoCA, CDR, HAMA, and HAMD) and sleep quality assessment scales (PSQI, ISI), before undergoing fMRI examination. HC met the following criteria: (1) no complaints of sleep disorders: PSQI score <5 and ISI score <7; (2) no neurological or psychiatric disorders, such as stroke, depression, and epilepsy etc.; (3) no neurological deficiency, such as visual or hearing loss; (4) no abnormal findings, such as infarction or focal lesion, on conventional brain MR images; (5) no cognitive complaints; (6) MMSE score ≥28; (7) MoCA score of 26; and (8) CDR score of 0.

Exclusion criteria for CID participants, determined by sleep monitoring and clinical examination, were as follows: (1) obstructive sleep apnea; (2) moderate to severe periodic limb movement during sleep (PLMS, total PLM index >25/h); (3) circadian rhythm sleep disorder determined by sleep-wake cycles of sleep (such as work time at day and night alternation); (4) severe general medical disorders of cardiovascular, endocrine, renal, or hepatic systems; (5) neurological disorders associated with potential cognitive dysfunction, including local brain lesions, traumatic brain injury with loss of consciousness or confusion, and dementia associated with neurosyphilis, parkinsonism, or Lewy body disease; (6) psychiatric disorders, including depression (HAMD > 35), anxiety (HAMA > 29), and alcohol or drug abuse; (7) MRI data quality does not meet the requirements.

We recruited 59 patients with CID and 30 HC. In the CID group, five participants were excluded because they were diagnosed with obstructive sleep apnea by PSG; three were excluded because they were diagnosed with restless legs syndrome; five were excluded as they were found to have cerebrovascular disease; five were excluded because of depression and anxiety; and two were excluded because of head motion causing poor imaging quality. In the HC group, two participants were excluded because of either cerebrovascular disease or head motion (larger than 2.5 mm translation in any axis and larger than 2.5° angular rotation in any axis, and mean point-to-point translation or rotation of more than 0.15 mm or 0.1°). Finally, 39 patients with CID and 28 HC qualified for the study. Table 1 presents the demographic, neuropsychological, and sleep characteristics of enrolled participants.

**Table 1:** **Demographic, clinical, neuropsychological, and PSG data for HC and patients with CID**.

	**HC (*n* = 28)**	**CID (*n* = 39)**	***p*****-value**
Gender (M/F)	5/23	6/33	0.09
Age (year)	51.25 ± 12.47	52.21 ± 11.74	0.75
Education (year)	14.07 ± 2.69	13.23 ± 3.38	0.28
MMSE	28.55 ± 0.45	26.91 ± 0.38	0.01
MoCA	26.37 ± 0.65	22.56 ± 0.55	<0.001
CDR	0	0.21 ± 0.27	<0.001
PSQI	0.37 ± 0.44	13.19 ± 0.37	<0.001
ISI	0.59 ± 0.62	15.52 ± 0.52	<0.001
HAMA	0.19 ± 0.7	9.61 ± 0.59	<0.001
HAMD	0.36 ± 0.46	8.53 ± 0.39	<0.001
**PSG**			
Total sleep time (min)	–	345.6 ± 40.5	–
Sleep-onset latency (min)	–	156.0 ± 24.8	–
Wake times	–	6.2 ± 2.3	–
NREM SWS (S3+S4)%	–	10.3 ± 2.1	–
REM%	–	15.6 ± 6.3	–
**HEAD ROTATION PARAMETERS**			
Mean translation (mm)	0.048 (0.027)	0.047 (0.024)	<0.001
Mean rotation (degree)	0.043 (0.021)	0.048 (0.027)	<0.001
Mean frame displacement	0.123 (0.058)	0.127 (0.065)	<0.001

*Chi-squared test was used for gender comparisons; two-tailed two-sample t-tests were used for age and education comparisons; ANCOVA was used for MMSE, MoCA, CDR, PSQI, ISI, HAMA, and HAMD comparisons, with age as a covariate; NREM SWS% = (S3 + S4) sleep time/total sleep time, %; REM%, REM duration/total sleep time, %*.

### Study procedure

One week before the study, enrolled patients with CID were instructed not to drink alcohol or caffeinated beverages until the end of study, and were asked to keep sleep diaries. The day before the study, patients arrived at the sleep monitoring center at 12:00 p.m. to acquaint themselves with the environment, and slept there from 12:30 to 2:00 p.m. Most patients with insomnia refused two consecutive nights of sleep monitoring. Therefore, we chose this approach as a compromise to avoid the first night effect.

On the day of formal sleep monitoring, participants completed a series of neuropsychological assessments from 6 to 7:30 p.m., including the MMSE, MoCA, CDR, HAMA, and HAMD. Subsequently, rs-fMRI data was acquired from 7:30 to 8 p.m., which could help avoid the impact of sleep status on data according to their sleep diaries (Tagliazucchi and Laufs, 2014; Stoffers et al., 2015). After the MRI scan, participants completed their sleep quality assessment scales, including the PSQI and ISI. Finally, they began the overnight monitoring with PSG.

### Sleep monitoring

Sleeping monitoring was conducted in a comfortable room, with a noise level below 30 decibels, good air ventilation, and a stable temperature at 20°C to help patients relax and reduce nervousness. Formal sleep monitoring began at 9:00 p.m. on the second day, and finished on the morning of the third day, with a total of ~7–9 h. The time of starting and ending sleep monitoring for participants was close to what they recorded in their sleep diary in the previous week.

Patients were asked to wash, work, and rest according to their normal sleep-wake cycle. Daytime sleep was prohibited and participants were asked to drink less water than usual to reduce nighttime urination. Sleep monitoring was recorded using a polysomnography (PSG; Remlogic, Embla Systems, Denver, CO, USA). The method of operation is designed according to the American Academy of Sleep Medicine (AASM) standards, including seven EEG leads (F3, F4, C3, C4, Cz, O1, and O2, connection mastoid), bilateral electrooculogram (EOG), mandibular and anterior tibial electromyography (EMG), and electrocardiogram (ECG) and respiratory rhythm monitoring (nose and mouth airflow, chest and abdominal breathing efficiency, and oxygen saturation). Sleep time was analyzed and calculated by an experienced technician (X.G.) from visual inspection and was reviewed by a neurologist (J.W.).

We collected data for total sleep time (TST), sleep-onset latency (SOL), wake time (WT), NREM SWA (S3 + S4) time and latency, and REM sleep time and latency. Before starting the experiment, participants were tested to confirm the EEG electrode connections and avoid ECG interference. After starting the experiment, healthcare professionals checked sleep monitoring devices every 30 min, including checking whether electrodes were peeling or oxygen saturation had changed.

### Neuropsychological assessment

Participants underwent a battery (1.5 h) of cognitive and psychological tests. MoCA scores were computed from eight measures, including visual spare, executive function, naming, memory, attention, language, abstract reasoning, and delayed recall. According to DSM-5 diagnostic criteria and the literature, diagnosis of anxiety, and depression disorders were derived from an interview to obtain HAMA and HAMD scores. A neuropsychologist evaluated all measurements according to Petersen standards, using the results of the assessments combined with clinical records of age and level of education. Furthermore, diagnosis of language function defect with mild cognitive impairment or delayed memory decline was made using MoCA scores.

### Self-report questionnaires about sleep

The Pittsburgh Sleep Quality Index (PSQI) measures sleep quality and disturbances during the past month. It is derived from the answers to nine questions and reflects seven problems of sleep quality (duration, subjective sleep quality, latency, habitual sleep efficiency, sleep disturbances, use of sleeping medication, and daytime dysfunction). A global score larger than five indicates poor sleep quality (Buysse et al., 1989; Ong et al., 2011). The ISI assesses a person's insomnia symptoms. A higher score represents more severe insomnia, with scores above 14 indicating a diagnosis of insomnia (Bastien et al., 2001; Morin et al., 2011).

### MRI acquisition

The MRI scan was performed with a 1.5 Tesla superconductor MRI scanner (Intera Achieva, Philips). Resting state-fMRI was performed using an echo planar imaging (EPI) sequence with the following parameters: TR/TE = 3,000/30 ms, flip angle = 90°, matrix = 64 × 64, field of view = 220 × 220 mm^2^, slice thickness = 3.6 mm with 1 mm slice gap. Each volume comprised 35 axial slices, and each functional scan lasted for 5 min and 6 s. Sagittal T1-weighted images (T1WI) were acquired continuously using three-dimensional magnetization with rapid gradient echo for three-dimensional reconstruction of spatial registration. The parameters were as follows: TR/TE = 1,900/2.2 ms, flip angle = 9°, inversion time = 900 ms, matrix = 256 × 256, field of view = 240 × 240 mm^2^, 1 mm slice thickness without slice gap. During scanning, participants were instructed to keep their eyes closed and to relax. Comfortable foam padding was used to minimize head motion and ear plugs were used to reduce noise.

Patients were asked to remain awake during scanning, and they were checked for whether they were asleep after the scan. If they were asleep, we repeated the scan. Although we could not complete synchronous EEG and eye movement detection during rs-fMRI scanning (about 15 min) due to limited experimental conditions, all participants claimed they had been awake. For each participant, MRI images were evaluated by two senior neuroradiologists to confirm no abnormalities were present.

### MRI data preprocessing

The fMRI data were preprocessed using a method consistent with protocols of previously published studies with the Brainnetome fMRI toolkit (Brant, http://www.brainnetome.org/en/brat). The preprocessing steps included: (1) slice-timing; (2) realignment to reduce head motion; (3) normalization to a standard EPI template and re-slicing to 2 × 2 × 2 mm cubic voxels; (4) de-noising by regressing out several effects (six motion parameters, linear drift, and the mean time series of all voxels within the white matter and cerebrospinal fluid); and (5) temporal filtering (0.01–0.08 Hz) to reduce noise. It has been showed that even small head motion during fMRI scanning will have a substantial impact on some measurements of resting-state fMRI (Power et al., 2012; Satterthwaite et al., 2012; Van Dijk et al., 2012). We have evaluated group differences in head motion among the two groups for the mean translation, mean rotation, and mean frame displacement and the results showed that the two groups had no significant differences in head motion (two-tailed two-sample *t*-test, *P* < 0.001; Table 1). The brain regions and their abbreviations used in this paper are shown in Table S1.

### Defining the connectivity nodes and estimation of interregional functional connectivity

Registered normalized fMRI volumes were partitioned into 116 regions (45 regions per hemisphere, and 26 cerebellar regions, as shown in Table S2) using an automated anatomical labeling template (Tzourio-Mazoyer et al., 2002) that has been used in several previous studies (Achard et al., 2006; Liu et al., 2007, 2008a; Salvador et al., 2007; Supekar et al., 2008; Zhao et al., 2012). For each sample, the representative time series of each brain region was obtained by averaging the fMRI time series over all voxels in the region.

The regional mean time series were estimated by averaging the time series of all voxels in the region (Salvador et al., 2005a,b; Achard et al., 2006). Pearson's correlation coefficients were computed between each pair of brain regions for each subject. For subsequent statistical analysis, Fisher's r-to-z transformation was applied to improve the normality of the correlation coefficients (Liu et al., 2007, 2008a).

### Statistical analysis

Individual *z*-scores were compared using a two-tailed one-sample *t*-test to determine whether two brain regions had significant functional connectivity within each group. For each region, a two-tailed two-sample *t*-test was used to determine whether there was a significant difference in functional connectivity between the CID and HC groups. A two-tailed two-sample *t*-test was performed for all 6,670 (116 × 115/2) functional connections. Thus, a correction for multiple comparisons was highly necessary.

In this study, we identified significant differences in functional connectivity between patients with and HC according to the following two criteria: (1) *z*-values were significantly different from zero in at least one group with *p* < 0.01 (two-tailed one-sample *t*-test; FDR corrected); (2) z-scores were significantly different between the two groups (two-tailed two-sample *t*-test, *p* < 0.01, permutation test corrected). To test the null hypothesis that the observed group differences could occur by chance, we randomly selected a number of HC, with the remaining HC as a second group and then calculated the group difference between these two randomized groups. This randomization procedure was repeated 10,000 times and then each distribution was used as the critical value for a one-tailed test of the null hypothesis, with a probability of type I error of 0.01. The procedure was repeated for each pair of regions with identified connectivity.

For the regions of brain connectivity identified as differing between patients with CID and HC, we used Pearson's correlation coefficient to evaluate any relationships between neuropsychological scores and brain functional connectivity strength across all patients. Because these analyses were exploratory in nature, we used a statistical significance level of *P* < 0.05 (uncorrected). In order to check if there exist any significant correlation, we also performed the false discovery rate (FDR) to control type 1 error in the context of testing correlations between MMSE scores and functional connectivity measures (*P* < 0.05).

## Results

### Demographic information and neuropsychological data, and correlations between variables

The present study included 39 patients with CID (male, *n* = 6) with a mean age of 52.2 ± 11.7 years and 28 HC (male, *n* = 5), who were age-, gender-, and education level-matched with patients. We observed significant differences in MMSE and MoCA scores between HC and CID, covarying for age. Patients with CID also had differences visible on PSG, including less TST (average 5.8 h), longer SOL (average 2.6 h), more WTs (6.2), and shorter NREM SWS (S3 + S4) and REM time. These results are consistent with two previous reports in the literature (Table 1; Bumb et al., 2014; Regen et al., 2016).

PSQI scores were correlated with MMSE and MoCA scores, with age, gender, and years of education as covariates (*r*_MMSE_ = −0.364, *p*_MMSE_ = 0.003; *r*_MoCA_ = −0.509, *p*_MoCA_ < 0.001).

### Functional connectivity of the whole brain

**Figure 1:**
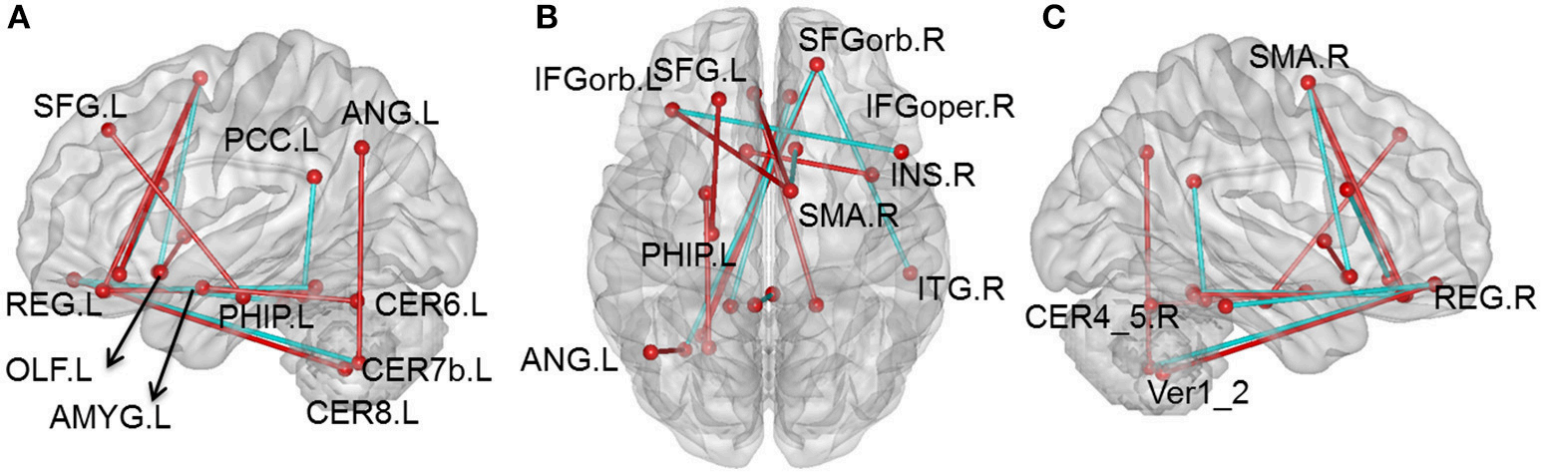
**Altered whole-brain connectivity patterns in the CID group compared with the HC group. (A–C)** Three-dimensional representation of the functional connectivity, and most of the affected nodes *p* < 0.001 (permutation *t*-test *p* < 0.001) in CID. Red lines represent decreased functional connectivity strength in CID, blue lines represent increased functional connectivity strength in CID. Details can be found at Table S1.

Compared with the HC group, there were 105 pairs of significantly different functional connectivity in patients with CID, including 78 pairs of positive correlations and 27 negative correlations (*t*-values are shown in Figure S1, Tables S2, S3 in Supplemental Material, *p* < 0.01, permutation corrected). Sixteen pairs of brain regions had extremely significantly altered functional connectivity at *p* < 0.001 (permutation corrected; as shown in Figure 1).

#### Altered positive correlations

Compared with the HC group, 76 positive correlations showed a significant decrease in CID (details shown in Table S2). Most of these were regions in the prefrontal lobe (61 pairs), while others were observed in various cortical and subcortical regions (cerebellum, temporal lobe, limbic lobe, and basal nuclei), and within the frontal lobe. There were also some decreases in positive correlations in the cingulate gyrus (8 pairs), including connections of the cingulate gyrus with the cerebellum and limbic lobe. In addition, we also found some decreases in positive correlations between the cerebellum and other cortical regions (7 pairs). Nearly half of these decreases occurred in key nodes of the DMN, including the internal DMN, the medial prefrontal cortex (mPFC), posterior cingulated cortex (PCC), and cerebellum. The 16 pairs of brain regions showing extremely significant differences were also mainly located between key DMN nodes (mPFC and PCC) and the cerebellum.

There was increased correlations between the left superior frontal gyrus (SFG) and the ipsilateral parahippocampal (PHIP), and between the left orbital middle frontal gyrus (MFGorb) and ipsilateral middle temporal gyrus (MTG; Table S2, *P* < 0.01, permutation *t*-test).

#### Altered negative correlations

There were 27 negative correlations that were significantly different between patients with CID and HC. As shown in Table S3, 21 negative correlations were significantly increased (apart from zero) in patients with CID. Most of these negative correlations were between the frontal lobe and other brain regions, including mPFC and MTG, superior parietal gyrus (SPG), dorsolateral prefrontal gyrus (dPFG), opercular inferior frontal gyrus (IFG), precuneus, inferior temporal gyrus (ITG), cerebellum and supplementary motor area (SMA), and cerebellum and amygdale. In addition, there were six decreased negative correlations between the temporal lobe and other brain regions (MTG-orbital frontal part, thalamus-Heschl, and fusiform-cerebellum VII).

### Relationships between sleep quality, clinical cognitive variables, and functional connectivity strength

**Figure 2:**
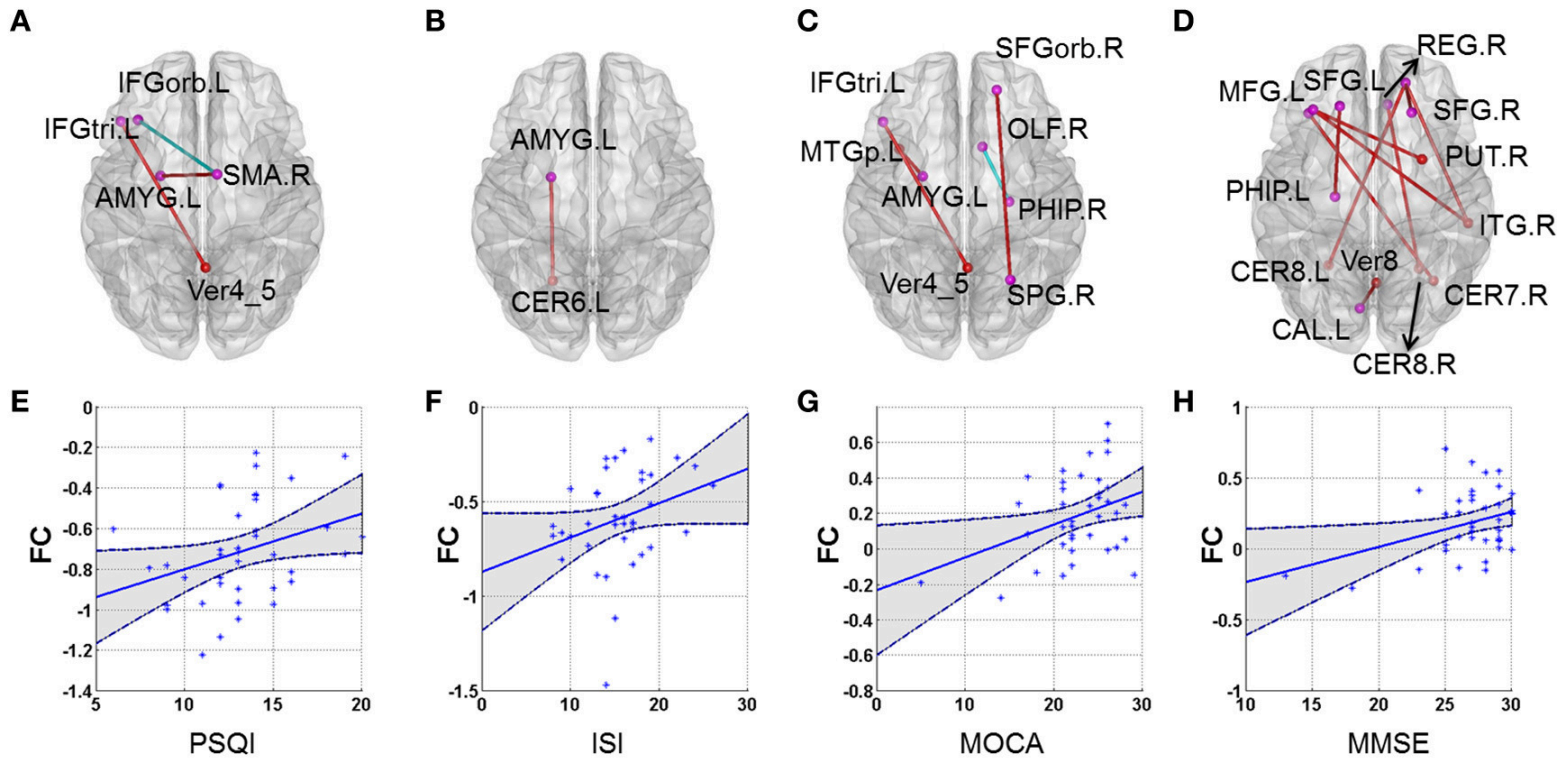
**Correlations between altered whole-brain connectivity patterns and subjective sleep scores and cognitive scores in the CID group. (A–D)** represent the correlation between functional connectivity and PSQI, ISI, MoCA, and MMSE, respectively. Red lines indicate positive correlations and blue lines indicate negative correlations. **(E)** Scatterplot showing a significant correlation between patient PSQI scores and functional connectivity in the left IFGorb and right SMA (*r* = −0.356, *p* = 0.026). **(F)** Scatterplot showing a significant correlation between patient ISI scores and functional connectivity in the left AMYG and left CER6 (*r* = 0.323, *p* = 0.045). **(G)** Scatterplot showing a correlation between patient MoCA scores and functional connectivity in the left IFG_inf_triangle and Verims_4_5 (*r* = 0.364, *p* = 0.023). **(H)** Scatterplot showing a significant correlation between patient MMSE scores and functional connectivity in the right SFGorb and right ITG (*r* = 0.464, *p* = 0.003). **(E–H)** just provide several examples for the behavioral-connection relationships. Details can be found in Table S1.

Our results showed that several of the functional connections identified as altered in patients with CID were correlated with sleep ratings in this group, although no significant correlation could survive the stringent FDR correction. The strength of the decreased positive correlation between the left orbital inferior frontal gyrus (IFGorb) and the right SMA was negatively correlated with PSQI ratings (*r* = −0.35, *p* = 0.031). The strength of the increased negative correlation between left amygdale and left cerebellum IX was positively correlated with ISI ratings (*r* = 0.32, *p* = 0.045; Table 2 and Figure 2).

**Table 2:** **Correlations between patient MMSE, ISI, and PSQI scores and strength of functional connectivity (FC; *p* < 0.05) among regions of impaired functional connectivity in CID**.

**Brain region 1**	**Brain region 2**	**MMSE-r**	**MMSE-p**	**MoCA-r**	**MoCA-p**	**ISI-r**	**ISI-p**	**PSQI-r**	**PSQI-p**
Frontal_Inf_Orb_L	Supp_Motor_Area_R	0.0872	0.5976	0.2157	0.1873	0.0015	0.9927	−**0.3453**	**0.0313**
Frontal_Sup_Orb_R	Cerebelum_8_L	**0.346**	**0.0309**	0.2022	0.2171	0.13	0.4302	−0.1008	0.5416
Frontal_Sup_L	ParaHippocampal_L	**0.4082**	**0.0099**	0.1062	0.52	0.0245	0.8825	0.1755	0.2852
Frontal_Sup_Orb_R	Temporal_Inf_R	**0.4639**	**0.0029**	0.2096	0.2003	0.0211	0.8988	0.1049	0.5252
Amygdala_L	Cerebelum_6_L	−0.142	0.3884	0.0109	0.9475	**0.3226**	**0.0452**	0.0284	0.8639
Rectus_R	Cerebelum_8_R	**0.3587**	**0.0249**	−0.0955	0.563	−0.1145	0.4875	−0.1593	0.3327
Frontal_Inf_Orb_L	Cerebelum_7b_R	**0.3532**	**0.0274**	0.0569	0.731	0.1165	0.4802	−0.1603	0.3297
Calcarine_L	Vermis_8	**0.355**	**0.0266**	−0.1004	0.5432	0.1096	0.5067	0.0687	0.6778
Frontal_Mid_L	Temporal_Inf_R	**0.4187**	**0.008**	−0.0201	0.9031	−0.1747	0.2876	0.1245	0.4501
Frontal_Sup_Orb_R	Parietal_Sup_R	0.2851	0.0785	**0.3308**	**0.0397**	0.1811	0.27	0.1174	0.4765
Frontal_Sup_R	Frontal_Sup_Orb_R	**0.4633**	**0.003**	0.2483	0.1275	−0.0228	0.8904	−0.0566	0.7321
Olfactory_R	ParaHippocampal_R	−0.1057	0.5218	−**0.3414**	**0.0334**	−0.0592	0.7204	−0.1809	0.2704
Frontal_Inf_Tri_L	Vermis_4_5	0.2702	0.0962	**0.3639**	**0.0228**	0.0395	0.8113	**0.317**	**0.0493**
Supp_Motor_Area_R	Amygdala_L	0.0941	0.5686	0.1116	0.4986	0.2586	0.112	**0.3556**	**0.0263**
Frontal_Mid_L	Putamen_R	**0.3197**	**0.0473**	0.0614	0.7104	0.0545	0.7418	0.1165	0.4801
Amygdala_L	Temporal_Pole_Mid_L	0.1609	0.3279	**0.3177**	**0.0488**	0.1535	0.3507	0.0025	0.9882

*MMSE-r, correlation coefficient between MMSE and the strength of functional connectivity; MMSE-p, p-value of the correlation coefficient between MMSE and the strength of functional connectivity; MoCA-r, correlation coefficient between MMSE and the strength of functional connectivity; MoCA-p, p-value of the correlation coefficient between MMSE and the strength of functional connectivity; ISI-r, correlation coefficient between MMSE and the strength of functional connectivity; ISI-p, p-value of the correlation coefficient between MMSE and the strength of functional connectivity; PSQI-r, correlation coefficient between MMSE and the strength of functional connectivity; PSQI-p, p-value of the correlation coefficient between MMSE and the strength of functional connectivity. The bold values means the significant correlation between patient MMSE, MoCA, ISI, and PSQI scores and strength of functional connectivity (p < 0.05)*.

Several functional connections were also correlated with cognitive scores, including the strength of the decreased positive correlation between the left triangular IFG and vermis4_5, which was positively correlated with MoCA scores. Furthermore, the strengths of the increased negative correlation between the right orbital superior frontal gyrus (SFGorb) and ipsilateral ITG, of the decreased positive correlation between the right SFGorb and left cerebellum, and of the increased positive correlation between the left SFG and ipsilateral PHIP were positively correlated with MMSE scores (*r* = 0.36, 0.46, 0.35, and 0.41, respectively, *p* < 0.05; Table 2 and Figure 2).

## Discussion

Previously reported analyses of brain functional connectivity in CID have been performed with region of interest (ROI) or regional homogeneity (ReHo) methods. In contrast, this study is the first to examine abnormal functional connectivity throughout the whole brain using resting-state fMRI. In addition to positive correlations between brain regions, we also considered abnormal negative correlations, which expands the breadth of knowledge in this field. Furthermore, we provide the first analysis of the relationship of whole brain functional connectivity with subjective insomnia scores and cognitive scale scores in patients with CID.

As shown in Table 2, 21 pairs of regions with increased negative correlations were located in the prefrontal lobe. The template used in this study differs from that used in previous research (Fox et al., 2005; Fransson, 2005), and therefore we used two templates to identify the abnormal brain regions to compare the techniques. We also observed that nearly one third of the regions of increased negative correlations were located in the mPFC, MTG, SPG, opercular IFG, precuneus, and ITG. The other regions of increased negative correlations were between the mPFG and cerebellum, and between the SMA and cerebellum. Previous studies have identified the key nodes of the DMN (the mPFC and precuneus), and the SPG, ITG, and cerebellar vermis as the task-negative network (Fox et al., 2005), while the IFG, MTG, dPFC, and SMA form the task-positive network. Combined with the results in Table 2, we have demonstrated increased negative correlation mainly located within the task-negative network and between the two intrinsic anti-correlation networks. It appears that CID leads to disruption of normal correlations within the task-negative network and between the two intrinsic anti-correlation networks.

Fox and colleagues have explained the relationships between regions within the anti-correlation network as mutually competitive (Fox et al., 2005), while Fransson suggested the two networks are alternately active, corresponding with different endogenous and exogenous attention states (Fransson, 2005). Based on this, we hypothesized that abnormal negative correlation might cause cognitive dysfunction in patients with CID because it interferes with these relationships. This viewpoint has also been confirmed by a recent animal study (Maingret et al., 2016). Interestingly, our results show that the enhanced negative correlation within the task-negative network, consisting of the right SFG and ipsilateral ITG, was positively correlated with MMSE scores. This is consistent with a study of working memory that reported decreased task-related brain activity with increasing task difficulty, as well as decreased regulated function of task-negative correlation regions (DMN key nodes; Drummond et al., 2013). Thus, our study demonstrates that abnormal correlations within the task-negative network itself and between two intrinsic anti-correlation networks, may lead to cognitive dysfunction in CID.

We also observed that positive correlation connectivity between several regions of the prefrontal lobe (61 pairs), and other cortical and subcortical regions was decreased in CID (Table S2). Previous structural and functional neuroimaging studies have demonstrated that the PFC is closely related to insomnia (Bell-McGinty et al., 2004; Altena et al., 2008, 2010; Lythe et al., 2012; Joo et al., 2013; Mander et al., 2013, 2015; Li et al., 2014; Verweij et al., 2014; Wilckens et al., 2016). Using structural MRI, functional MRI, PET, and polysomnography, Mander and colleagues found that gray matter atrophy and β-amyloid deposition in the PFC indirectly disrupts functional connectivity between the hippocampus and mPFC through the intermediary factor of NREM SWA, which may disrupt overnight consolidation of hippocampal-cortical dependent memory systems (Mander et al., 2013, 2015). Evidence suggests that decreased NREM SWA in healthy people is correlated with the prefrontal metabolic rate in states of arousal (Wilckens et al., 2016). All previous results have suggested that decreased NREM SWA would disrupt functional connectivity of the PFC. Using rs-fMRI, Vermeil and colleagues have observed that short-term sleep deprivation disturbs functional connectivity in the PFC, which suggests that sleep reserves play an important role in sustaining the function of this region (Verweij et al., 2014). Our results suggest that subjective sleep score is correlated with disrupted brain functional connectivity in patients with CID. For example, the connection strength between the left IFGorb and right SMA was negatively correlated with PSQI scores, i.e., the aggravation of insomnia. There were greater reductions in functional connectivity between the PFC and the SMA with increased insomnia, which further confirms that insomnia can disturb prefrontal functional connectivity.

We found that the most significant reductions in functional connectivity mainly occurred between key nodes of the DMN, the mPFC, and the PCC, including between mPFC (SFGorb, MFGorb, and IFGorb) and temporal lobe, cerebellum, limbic lobe, primary motor area and SMA, PCC, and cerebellum (Figure 1 and Table S2). This indicates that disrupted connectivity is mainly located within the DMN, key nodes of the DMN, and the cerebellum. Prior studies have also indicated DMN disconnection in insomnia (De Havas et al., 2012; Khalsa et al., 2016; Suh et al., 2016). Using fMRI and a visual attention task, De Havas and colleagues reported that after 24 h sleep deprivation, DMN intrinsic connectivity, and connectivity between two intrinsic anti-correlation networks was significantly reduced (De Havas et al., 2012). Applying cortical thinning covariance within the DMN, Suh and colleagues found that anterior and posterior regions of DMN are correlated with insomnia and cognitive impairment (Suh et al., 2016). In contrast, cumulative habitual total sleep time (cTST) could predict intrinsic functional connectivity of the DMN, and the correlation between the DMN and the salience network in healthy people. For instance, cTST is positively correlated with intrinsic connections of the DMN (Khalsa et al., 2016). These studies strongly suggest that sleep loss can cause DMN network disconnection.

Concurrently, another study has suggested that there is a disconnection between the frontal lobe and cerebellum in healthy people after sleep deprivation (Liu et al., 2015). Multiple methods, including genetics, electrophysiology, MRI and PET, have suggested that sleep quality is associated with the pathophysiology, morphology, and function of the cerebellum (Mano, 1970; Bell-McGinty et al., 2004; Howell et al., 2006; Joo et al., 2013; Dai et al., 2014; Wu et al., 2014; Liu et al., 2015). The cognitive function of healthy people depends on multiple sub-regions of the cerebellum (Sang et al., 2012). In healthy people, there is functional connectivity between the cerebellum II region and frontoparietal networks, and between the cerebellum IX region and the DMN, while patients with insomnia often show neuropathological indications of impaired connections in the frontoparietal network or DMN (De Havas et al., 2012; Li et al., 2014; Sexton et al., 2014; Khalsa et al., 2016; Suh et al., 2016). Our results suggest that CID accompanied by cognitive dysfunction may be associated with a disconnection between the DMN and posterior cerebellum. Previous fMRI studies of cognition have also confirmed that sleep loss has an important impact on the cerebellum and cognitive function. This could explain why the activity of the cerebellum decreased and recognition accuracy was reduced after short-term sleep deprivation (Bell-McGinty et al., 2004). Combined with the results of the current study, decreased strength of the positive correlation between the right SFGorb and left cerebellum VIII was positively correlated with MMSE scores. Therefore, we believe that disruption of the frontal-cerebellum loop is a potential reason for insomnia accompanied by cognitive dysfunction.

In the present study, we also identified two increased positive connections between the left SFC and ipsilateral PHIP, and between the left MFGorb and ipsilateral MTG (Table S2). Previous cross-sectional and longitudinal rs-fMRI studies have reported have increased functional connectivity in the prefrontal lobe and DMN in patients with mild cognitive impairment (Wang et al., 2007, 2012; Agosta et al., 2012). The PHIP is involved in regulating connectivity between the hippocampus and the DMN, and is related to information processing for various cognitive functions (Ward et al., 2014). Meanwhile, the increased connectivity between the left SFG and ipsilateral PHIP is positively correlated with MMSE scores, suggesting that functional connectivity of the frontal-temporal cortex could modify cognitive function after prefrontal disconnection.

Considering our results, increased negative correlations are mainly located in the intrinsic task-negative network, and between the two intrinsic anti-correlation networks, while decreased positive correlation was observed in the intrinsic DMN and between key nodes of the DMN and the cerebellum. Furthermore, the functional connection between the prefrontal lobe (61 pairs) and other brain region is related to the aggravation of insomnia. Therefore, we believe that the disruption within the task-negative network and the abnormal correlation between the two intrinsic anti-correlation networks, may represent an important pathological mechanism of CID. More importantly, the increased negative correlation and the decreased positive correlation between brain regions may be positively correlated with patient MMSE scores. In consequence, we further infer that abnormal correlation in the intrinsic task-negative network, and between the two intrinsic anti-correlation networks, may be important neurobiological indicators of CID and accompanying cognitive impairment.

This study is subject to some limitations that should be borne in mind when interpreting the results. The main limitation was that we did not conduct an objective sleep test and subjective sleep scores were negative in healthy participants, so we only analyzed brain network changes in CID and its correlation with subjective sleep scores. Further, studies are needed to improve sleep monitoring indicators, such as NREM SWS and REM sleep duration, sleep latency, and band characteristics, as well as analyzing correlations between electrophysiology and fMRI functional connectivity. In addition, we analyzed altered brain connectivities in the insomnia group and their correlation with sleep quality, clinical cognitive variables. We identified several functional connectivities associated with poor sleep quality and/or declined cognitive ability. However, the analysis could not completely avoid the type I error for these results did not survive the FDR correction. One possible reason is the small sample size is not large enough to give sufficient statistical power to identify significant effects. Further, studies are needed with larger sample sizes that analyze the brain function changes in insomnia with or without cognitive impairment. Such studies may be an important step in revealing the brain network mechanisms of insomnia accompanied by cognitive impairment.

## Conclusions

Our results provide new evidence for a disconnection between prefrontal cortex and the DMN in insomnia as reported in previous studies. In addition, we discovered the presence of negative correlations that were not reported in the literature. Our results imply that the abnormal correlation in the intrinsic task-negative network and between the two intrinsic anti-correlation networks may be important neurobiological indicators of CID and accompanying cognitive impairment. Overall, our study describes the whole brain connections of CID, and provides an initial description of the brain network mechanisms underlying CID with cognitive impairment, which provides a foundation for future related studies.

## Ethics statement

This study was carried out in accordance with the recommendations of “the Committee on Human Experimentation of the institution in which the experiments were done or in accord with the Helsinki Declaration of 1975” with written informed consent from all subjects. This experiment was conducted on humans. All experimental protocols were approved by Clinical Research Ethics Committee of Dongfang Hospital of Beijing University of Chinese Medicine. The methods were carried out in accordance with the approved guidelines. Informed consent was obtained from all participants before participation.

## Author note

The work was designed at Xuanwu Hospital of Capital Medical University, and was performed at Dongfang Hospital of Beijing University of Chinese Medicine.

## Author contributions

RP, YuZ, RG, JW, XG collected the data. YL, YaZ analyzed data and obtained the measurements. RP wrote the main text and YL prepared Figures 1, 2. ZW revised the important intellectual content of the manuscript. KL provided final approval of the version to be published and supervised the project to ensure that questions related to the accuracy or integrity of any part of the work were appropriately investigated and resolved. All authors reviewed the manuscript and approved it for publication.

## Funding

This work was partially supported by the National Key Research and Development Program of China (No. 2016YFC1305904) and National Natural Science Foundation of China (Grant Nos. 81471649, 81571648, 81370037, 81571062).

### Conflict of interest statement

The authors declare that the research was conducted in the absence of any commercial or financial relationships that could be construed as a potential conflict of interest.

## Supplementary material

Table S1 **Cortical and subcortical regions defined in Automated Anatomical Labeling template image in standard stereotaxic space**.

Table S2 **The positive interregional correlation in CID patients compared with healthy controls**.

Table S3 **The negative interregional correlation in CID patients compared with healthy controls**.

Figure S1 **Altered whole-brain connectivity patterns in the CID group compared with the normal control group**.

## Abbreviations

AD: Alzheimer's disease
AASM: American Academy of Sleep Medicine
AAL: automated anatomical labeling
CID: chronic insomnia disorder
CDR: Clinical Dementia Rating
cTST: cumulative habitual total sleep time
DMN: default mode network
dPFG: dorsolateral prefrontal gyrus
ECG: electrocardiogram
EEG: electroencephalograph
EMG: electromyography
EOG: electrooculogram
EPI: echo planar imaging
DSM-5: Diagnostic and Statistical Manual of Mental Disorders-Fifth edition
HAMA: Hamilton Anxiety Scale
HAMD: Hamilton Depression Scale
HC: healthy controls
ISI: Insomnia Severity Index
ITG: inferior temporal gyrus
MMSE: Mini-Mental State Examination
MoCA: Montreal Cognitive Assessment
mPFC: medial prefrontal cortex
MTG: middle temporal gyrus
NREM: non-rapid eye movement
IFGorb: orbital inferior frontal gyrus
MFGorb: orbital middle frontal gyrus
PCC: posterior cingulate cortex
PHIP: parahippocampal
PSG: polysomnogram
PSQI: Pittsburgh Sleep Quality Index
rs-fMRI: Resting-state fMRI
SWS: slow wave activity
SFG: superior frontal gyrus
SMA: supplementary motor area
SOL: sleep-onset latency
SPG: superior parietal gyrus
ICSD-3: International Classification of Sleep Disorders-Third Edition
TST: total sleep time
T1WI: T1-weighted images
WT: wake time.
