# Supplementary material for: Aberrant Functional Connectivity Architecture in Participants with Chronic Insomnia Disorder Accompanying Cognitive Dysfunction: A Whole-Brain, Data-Driven Analysis
Source: Front Neurosci. 2017 May 11;11:259. doi: 10.3389/fnins.2017.00259 (PMC5425485; doi:10.3389/fnins.2017.00259)
Supplement: Table S1 — Cortical and subcortical regions defined in Automated Anatomical Labeling template image in standard stereotaxic space. [file Table1.DOC]

# Table S1.

Table S1. Cortical and subcortical regions defined in Automated Anatomical Labeling template image in standard stereotaxic space

| Region Name | Abbreviation | MNI coordinates (L/R) |
| --- | --- | --- |
| **Prefrontal Lobe** |  |  |
| Superior frontal gyrus, dorsolateral | SFGdor | (-17,47,-13)/ (18,48,-14) |
| Superior frontal gyrus, orbital | SFGorb | (-5,49,31)/ (9,51,30) |
| Superior frontal gyrus, medial | SFGmed | (-5,54,-7)/ (8,52,-7) |
| Superior frontal gyrus, medial orbital | SFGmorb | (-33,33,35)/ (38,33,34) |
| Middle frontal gyrus | MFG | (-31,50,-10)/ (33,53,-11) |
| Middle frontal gyrus, orbital | MFGorb | (-48,13,19)/ (50,15,21) |
| Inferior frontal gyrus, opercular | IFGoper | (-46,30,14)/ (50,30,14) |
| Inferior frontal gyrus, triangular | IFGtri | (-36,31,-12)/ (41,32,-12) |
| Inferior frontal gyrus, orbital | IFGorb | (-5,37,-18)/ (8,36,-18) |
| Gyrus rectus | REG | (-4,35,14)/ (8,37,16) |
| Anterior cingulate gyrus | ACC | (-8,15,-11)/ (10,16,-11) |
| Olfactory cortex | OLF | (-18,35,42)/ (22,31,44) |
| **Other Prefrontal Lobe** |  |  |
| Precentral gyrus | PreCG | (-39,-6,51)/ (41,-8,52) |
| Supplementary motor area | SMA | (-5,5,61)/ (9,0,62) |
| Rolandic operculum | ROL | (-47,-8,14)/ (53,-6,15) |
| Median- and para-cingulate gyrus | MCC | (-5,-15,42)/ (8,-9,40) |
| **Occipital Lobe** |  |  |
| Calcarine fissure and surrounding cortex | CAL | (-7,-79,6)/ (16,-73,9) |
| Cuneus | CUN | (-6,-80,27)/ (14,-79,28) |
| Lingual gyrus | LING | (-15,-68,-5)/ (16,-67,-4) |
| Superior occipital gyrus | SOG | (-17,-84,28)/ (24,-81,31) |
| Middle occipital gyrus | MOG | (-32,-81,16)/ (37,-80,19) |
| Inferior occipital gyrus | IOG | (-36,-78,-8)/ (38,-82,-8) |
| Fusiform gyrus | FG | (-31,-40,-20)/ (34,-39,-20) |
| **Parietal Lobe** |  |  |
| Superior parietal gyrus | SPG | (-23,-60,59)/ (26,-59,62) |
| Paracentral lobule | PCL | (-7,-56,48)/ (10,-56,44) |
| Postcentral gyrus | PoCG | (-42,-23,49)/ (41,-25,53) |
| Inferior parietal gyrus | IPG | (-43,-46,47)/ (46,-46,50) |
| Supramarginal gyrus | SMG | (-56,-34,30)/ (58,-32,34) |
| Angular gyrus | ANG | (-44,-61,36)/ (46,-60,39) |
| Precuneus | PCNU | (-8,-25,70)/ (7,-32,68) |
| Posterior cingulate gyrus | PCC | (-5,-43,25)/ (7,-42,22) |
|  |  |  |
| **Insula** | INS | (-35,7,3)/ (39,6,2) |
| **Thalamus** | THA | (-11,-18,8)/ (13,-18,8) |
| **Temporal Lobe** |  |  |
| Superior temporal gyrus | STG | (-53,-21,7)/ (58,-22,7) |
| Superior temporal gyrus, temporal pole | STGp | (-40,15,-20)/ (48,15,-17) |
| Middle temporal gyrus | MTG | (-56,-34,-2)/ (57,-37,-1) |
| Middle temporal gyrus, temporal pole | MTGp | (-36,15,-34)/ (44,15,-32) |
| Inferior temporal gyrus | ITG | (-50,-28,-23)/ (54,-31,-22) |
| Heschl gyrus | HES | (-42,-19,10)/ (46,-17,10) |
| Hippocampus | HIP | (-25,-21,-10)/ (29,-20,-10) |
| Parahippocampal gyrus | PHIP | (-21,-16,-21)/ (25,-15,-20) |
| Amygdala | AMYG | (-23,-1,-17)/ (27,1,-18) |
| **Basal Ganglia** |  |  |
| Caudate nucleus | CAU | (-11,11,9)/ (15,12,9) |
| Lenticular nucleus, putamen | PUT | (-24,4,2)/ (28,5,2) |
| Lenticular nucleus, pallidum | PAL | (-18,0,0)/ (21,0,0) |
| Cerebelum_Crus1 | CERcr1 | (35,-67,-29)/(-38,-67,-30) |
| Cerebelum_Crus2 | CERcr2 | (28,-73,-38)/(-33,-69,-40) |
| Cerebelum_Crus3 | CERcr3 | (8,-37,-19)/(-13,-34,-19) |
| Cerebelum_Crus4_5 | CERcr4_5 | (14,-43,-17)/(-18,-43,-18) |
| Cerebelum_Crus6 | CERcr6 | (22,-59,-22)/(-26,-58,-24) |
| Cerebelum_Crus7 | CERcr7 | (31,-60,-45)/(-34,-63,-48) |
| Cerebelum_Crus8 | CERcr8 | (25,-55,-48)/(-26,-56,-49) |
| Cerebelum_Crus9 | CERcr9 | (10,-49,-46)/(-10,-49,-46) |
| Cerebelum_Crus10 | CERcr10 | (22,-34,-42)/(-27,-34,-41) |
| Vermis_1_2 | Ver1_2 | (-2,-39,-20) |
| Vermis_3 | Ver3 | (-2,-40,-11) |
| Vermis_4_5 | Ver4_5 | (-2,-52,-6) |
| Vermis_6 | Ver6 | (-2,-67,-15) |
| Vermis_7 | Ver7 | (-2,-72,-25) |
| Vermis_8 | Ver8 | (-2,-64,-34) |
| Vermis_9 | Ver9 | (-2,-55,-35) |
| Vermis_10 | Ver10 | (-1,-46,-32) |
|  |  |  |

Note: The abbreviations listed are those used in this paper, which differ slightly from the original abbreviations by Tzourio-Mazoyer et al. . And the center of mass coordinates are the same with Liu et al. .

MNI: Montreal Neurological Institute.
