# Supplementary material for: Aberrant Functional Connectivity Architecture in Participants with Chronic Insomnia Disorder Accompanying Cognitive Dysfunction: A Whole-Brain, Data-Driven Analysis
Source: Front Neurosci. 2017 May 11;11:259. doi: 10.3389/fnins.2017.00259 (PMC5425485; doi:10.3389/fnins.2017.00259)
Supplement: Table S2 — The positive interregional correlation in CID patients compared with healthy controls. [file Table2.DOC]

# Table S2.

Table S2. The positive interregional correlation in CID patients compared with healthy controls

| ROI | ROI | ttest p | t-stats | mean NC | mean PI |
| --- | --- | --- | --- | --- | --- |
| Supp_Motor_Area_R | Rectus_L | 0.0003 | 3.7974 | 0.379 | 0.1572 |
| Supp_Motor_Area_R | Rectus_R | 0.004 | 2.9864 | 0.868 | 0.6925 |
| Frontal_Inf_Oper_R | Frontal_Inf_Orb_R | 0.0033 | 3.0516 | 0.7849 | 0.577 |
| Frontal_Inf_Tri_R | Frontal_Inf_Orb_L | 0.0023 | 3.1695 | 0.4264 | 0.2174 |
| Frontal_Inf_Tri_R | Frontal_Inf_Orb_R | 0.0074 | 2.7649 | 0.7215 | 0.4977 |
| Frontal_Sup_R | Rectus_L | 0.0029 | 3.0973 | 0.6579 | 0.4931 |
| Rectus_L | Cerebelum_3_R | 0.0056 | 2.8672 | 1.4185 | 1.2466 |
| Rectus_L | Cerebelum_4_5_L | 0.0029 | 3.096 | 0.7406 | 0.5428 |
| Rectus_L | Cerebelum_4_5_R | 0.0005 | 3.6714 | 0.6686 | 0.4393 |
| Rectus_L | Cerebelum_8_L | 0.0065 | 2.8145 | 0.6498 | 0.4824 |
| Rectus_R | Cerebelum_4_5_L | 0.0008 | 3.5353 | 0.3213 | 0.1311 |
| Rectus_R | Cerebelum_4_5_R | 0.0069 | 2.7886 | 0.5522 | 0.3791 |
| Rectus_R | Cerebelum_7b_R | 0.0043 | 2.9616 | 1.6734 | 1.5157 |
| Rectus_R | Cerebelum_8_L | 0.0005 | 3.6939 | 1.133 | 0.9127 |
| Rectus_R | Cerebelum_8_R | 0.0023 | 3.1762 | 1.5732 | 1.3454 |
| Rectus_R | Cerebelum_9_L | 0.0069 | 2.7904 | 0.4117 | 0.2426 |
| Frontal_Inf_Tri_L | Cerebelum_Crus1_L | 0.0081 | 2.7319 | 1.238 | 1.1138 |
| Frontal_Inf_Tri_L | Vermis_4_5 | 0.0062 | 2.8304 | 1.1803 | 1.0291 |
| Olfactory_L | Calcarine_L | 0.0027 | 3.1193 | 0.3554 | 0.1738 |
| Olfactory_R | ParaHippocampal_R | 0.006 | 2.8419 | 0.2431 | 0.065 |
| Rectus_L | Calcarine_L | 0.0069 | 2.7908 | 0.6917 | 0.5012 |
| Rectus_L | Calcarine_R | 0.0053 | 2.8882 | 1.1741 | 0.9803 |
| Rectus_R | Calcarine_R | 0.0068 | 2.7974 | 1.7058 | 1.5165 |
| Olfactory_R | Putamen_R | 0.0011 | 3.4035 | 0.5699 | 0.3237 |
| Rectus_L | Putamen_R | 0.0023 | 3.1763 | 0.5177 | 0.2915 |
| Rectus_R | Putamen_R | 0.0019 | 3.2419 | 1.1946 | 0.9607 |
| Frontal_Mid_L | Putamen_R | 0.008 | 2.7345 | 0.6866 | 0.5087 |
| Olfactory_R | Cingulum_Mid_R | 0.0071 | 2.7826 | 1.4905 | 1.3074 |
| Frontal_Inf_Oper_R | Cingulum_Ant_R | 0.0071 | 2.7803 | 0.5707 | 0.41 |
| Olfactory_R | Temporal_Mid_R | 0.0044 | 2.9478 | 0.6247 | 0.4701 |
| Frontal_Mid_L | Temporal_Inf_R | 0.0042 | 2.9656 | 0.8453 | 0.6343 |
| Olfactory_L | Insula_R | 0.0004 | 3.7267 | 0.9382 | 0.6976 |
| Olfactory_L | Precuneus_L | 0.0074 | 2.7633 | 0.1792 | 0.0436 |
| Rectus_R | Occipital_Sup_L | 0.0041 | 2.9768 | 1.074 | 0.8882 |
| Rectus_L | Thalamus_L | 0.0083 | 2.7207 | 0.5548 | 0.3506 |
| Frontal_Inf_Orb_L | Supp_Motor_Area_R | 0 | 4.4535 | 1.1214 | 0.8376 |
| Frontal_Inf_Orb_L | Paracentral_Lobule_L | 0.0026 | 3.1264 | 0.656 | 0.4423 |
| Frontal_Inf_Orb_R | Supp_Motor_Area_R | 0.0069 | 2.7918 | 0.3429 | 0.1634 |
| Frontal_Inf_Orb_L | Rolandic_Oper_R | 0.0063 | 2.8238 | 0.9544 | 0.807 |
| Frontal_Inf_Orb_L | Fusiform_L | 0.0017 | 3.2747 | 0.2868 | 0.0841 |
| Frontal_Inf_Orb_L | Temporal_Sup_L | 0.0045 | 2.9452 | 0.7897 | 0.6078 |
| Frontal_Inf_Orb_L | Temporal_Sup_R | 0.0038 | 3.006 | 0.669 | 0.4821 |
| Frontal_Inf_Orb_L | Temporal_Pole_Sup_R | 0.0011 | 3.416 | 0.5267 | 0.3259 |
| Frontal_Inf_Orb_L | Temporal_Inf_R | 0.0054 | 2.8755 | 0.7562 | 0.5562 |
| Frontal_Inf_Orb_R | Temporal_Sup_L | 0.0021 | 3.2019 | 0.7625 | 0.5427 |
| Frontal_Sup_Orb_R | Cerebelum_7b_L | 0.0004 | 3.7525 | 0.2898 | 0.0829 |
| Frontal_Sup_Orb_R | Cerebelum_8_L | 0.0009 | 3.4831 | 0.6292 | 0.4275 |
| Frontal_Mid_Orb_R | Vermis_4_5 | 0.0029 | 3.0942 | 0.3632 | 0.1983 |
| Frontal_Inf_Orb_L | Cerebelum_7b_R | 0.0025 | 3.1453 | 0.7758 | 0.5968 |
| Frontal_Inf_Orb_L | Cerebelum_8_L | 0.0034 | 3.0408 | 1.174 | 0.9625 |
| Frontal_Inf_Orb_L | Cerebelum_8_R | 0.0032 | 3.0622 | 0.7713 | 0.5733 |
| Frontal_Inf_Orb_R | Cerebelum_8_R | 0.0048 | 2.9225 | 0.6677 | 0.4676 |
| Frontal_Inf_Orb_L | Amygdala_R | 0.0049 | 2.9166 | 0.9372 | 0.7798 |
| Frontal_Sup_Orb_R | Calcarine_L | 0.001 | 3.4333 | 0.4035 | 0.2229 |
| Frontal_Inf_Orb_L | Calcarine_L | 0.0023 | 3.1726 | 0.6135 | 0.4251 |
| Frontal_Inf_Orb_L | Parietal_Sup_L | 0.009 | 2.6928 | 1.0452 | 0.8897 |
| Frontal_Inf_Orb_L | Parietal_Sup_R | 0.0087 | 2.7058 | 0.7867 | 0.6119 |
| Frontal_Inf_Orb_L | Cuneus_R | 0.0048 | 2.9185 | 0.8428 | 0.6525 |
| Frontal_Inf_Orb_R | Insula_R | 0.0028 | 3.1061 | 0.3906 | 0.1631 |
| Cingulum_Post_L | ParaHippocampal_R | 0.0042 | 2.9649 | 1.3895 | 1.2048 |
| Cingulum_Post_L | Amygdala_R | 0.0085 | 2.7153 | 0.8889 | 0.7457 |
| Cingulum_Post_L | Cerebelum_3_L | 0.0078 | 2.7463 | 0.725 | 0.557 |
| Cingulum_Post_L | Cerebelum_3_R | 0.0025 | 3.1417 | 0.6417 | 0.4506 |
| Cingulum_Post_L | Cerebelum_7b_R | 0.0068 | 2.7933 | 1.2955 | 1.162 |
| Cingulum_Post_L | Vermis_1_2 | 0.0004 | 3.7155 | 0.9708 | 0.7444 |
| Cingulum_Ant_R | Cerebelum_8_R | 0.0092 | 2.6843 | 1.7011 | 1.5373 |
| Cingulum_Ant_R | Temporal_Mid_R | 0.0018 | 3.2573 | 0.2222 | 0.0478 |
| Hippocampus_L | Occipital_Inf_R | 0.0045 | -2.9406 | -0.1583 |  |
| Amygdala_L | Temporal_Pole_Mid_L | 0.0097 | 2.6647 | 1.1679 | 0.9845 |
| Amygdala_R | Cerebelum_6_L | 0.0032 | 3.0654 | 0.6715 | 0.4997 |
| Amygdala_R | Cerebelum_6_R | 0.0068 | 2.7981 | 0.7809 | 0.6317 |
| Calcarine_L | Vermis_8 | 0.0034 | 3.0433 | 0.8655 | 0.7257 |
| Angular_L | Cerebelum_Crus2_L | 0.0066 | 2.8085 | 0.8473 | 0.6484 |
| Angular_L | Cerebelum_7b_L | 0.0002 | 4.0041 | 0.7737 | 0.54 |
| Angular_L | Cerebelum_8_L | 0.0015 | 3.3101 | 1.5983 | 1.4087 |
| Cerebelum_6_L | Vermis_8 | 0.0049 | 2.9139 | 1.6142 | 1.459 |
| Frontal_Sup_L | ParaHippocampal_L | 0.0006 | -3.633 | -0.0339 | 0.193 |
| Frontal_Mid_Orb_L | Temporal_Pole_Mid_L | 0.0068 | -2.7967 | 0.0438 | 0.24 |

Black typeface for reduced positive correlation region；Red typeface for increased positive correlation region；The shaded part represents the reduced positive correlation region which occurred in the DMN key nodes (mPFC, PCC)
