# Supplementary material for: Aberrant Functional Connectivity Architecture in Participants with Chronic Insomnia Disorder Accompanying Cognitive Dysfunction: A Whole-Brain, Data-Driven Analysis
Source: Front Neurosci. 2017 May 11;11:259. doi: 10.3389/fnins.2017.00259 (PMC5425485; doi:10.3389/fnins.2017.00259)
Supplement: Table S3 — The negative interregional correlation in CID patients compared with healthy controls. [file Table3.DOC]

# Table S3.

Table S3. The negative interregional correlation in CID patients compared with healthy controls

| ROI | ROI | ttest p | t-stats | mean HC | mean PI |
| --- | --- | --- | --- | --- | --- |
| Frontal_Sup_R | Frontal_Sup_Orb_R | 0.0049 | 2.9166 | -0.3216 | -0.5117 |
| Frontal_Sup_Orb_R | Cuneus_R | 0.0085 | 2.7135 | -0.223 | -0.3963 |
| Frontal_Sup_Orb_R | Fusiform_L | 0.0012 | 3.3786 | -0.6071 | -0.8163 |
| Frontal_Sup_Orb_R | Parietal_Sup_R | 0.0046 | 2.9374 | -0.2005 | -0.4045 |
| Frontal_Sup_Orb_R | Precuneus_R | 0.0023 | 3.1772 | -0.1167 | -0.2864 |
| Frontal_Sup_Orb_R | Temporal_Inf_R | 0.0002 | 3.9793 | -0.9377 | -1.1875 |
| Frontal_Sup_Orb_R | Cerebelum_Crus2_L | 0.0042 | 2.9701 | -0.0155 | -0.1805 |
| Frontal_Sup_Orb_R | Cerebelum_6_L | 0.0071 | 2.7786 | -0.47 | -0.6416 |
| Frontal_Sup_Orb_R | Cerebelum_6_R | 0.0075 | 2.7585 | -0.0039 | -0.1685 |
| Frontal_Inf_Orb_R | Temporal_Mid_L | 0.0083 | 2.7207 | -0.4692 | -0.6493 |
| Frontal_Inf_Orb_R | Temporal_Mid_R | 0.0077 | 2.7523 | -0.0827 | -0.2648 |
| Frontal_Inf_Orb_L | Frontal_Inf_Oper_R | 0.0006 | 3.6025 | -0.1991 | -0.4286 |
| Frontal_Inf_Orb_L | Occipital_Sup_L | 0.0032 | 3.061 | -0.2114 | -0.4124 |
| Frontal_Inf_Orb_L | Temporal_Mid_R | 0.0025 | 3.1492 | 0.0165 | -0.1695 |
| Frontal_Inf_Orb_L | Cerebelum_7b_L | 0.0032 | 3.0598 | -0.1397 | -0.3125 |
| Supp_Motor_Area_R | Olfactory_R | 0.0004 | 3.7616 | 0.0551 | -0.125 |
| Supp_Motor_Area_R | Amygdala_L | 0.0066 | 2.8082 | -0.5564 | -0.7131 |
| Supp_Motor_Area_R | Fusiform_L | 0.0043 | 2.9623 | 0.0055 | -0.1648 |
| Supp_Motor_Area_R | Cerebelum_6_L | 0.0069 | 2.7882 | 0.0084 | -0.1654 |
| Olfactory_R | Temporal_Sup_R | 0.0084 | 2.7191 | -0.0472 | -0.2219 |
| Amygdala_L | Cerebelum_6_L | 0.0002 | 3.9155 | -0.3678 | -0.5908 |
| Rectus_L | Temporal_Mid_L | 0.0049 | 2.9139 | 0.1288 | -0.0429 |
| Cingulum_Mid_L | Vermis_6 | 0.0016 | -3.3009 | -0.354 | -0.1501 |
| Fusiform_L | Cerebelum_7b_R | 0.005 | 2.9047 | 0.1345 | -0.0573 |
| Thalamus_R | Heschl_L | 0.0086 | -2.7087 | -0.7013 | -0.501 |
| Vermis_8 | Vermis_10 | 0.0013 | -3.3661 | -0.8544 | -0.687 |
| Olfactory_L | Temporal_Mid_R | 0.006 | 2.8431 | 0.122 | -0.0065 |

Black typeface for increased negative correlation region；Blue typeface for decreased negative correlation region
