# Supplementary figures and images for: Aberrant Functional Connectivity Architecture in Participants with Chronic Insomnia Disorder Accompanying Cognitive Dysfunction: A Whole-Brain, Data-Driven Analysis
Source: Front Neurosci. 2017 May 11;11:259. doi: 10.3389/fnins.2017.00259 (PMC5425485; doi:10.3389/fnins.2017.00259)

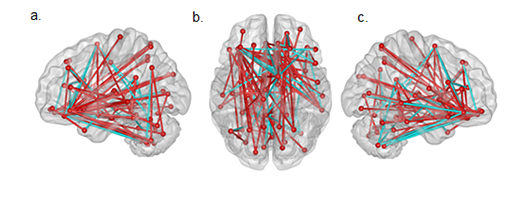

Supplement: Figure S1 — Altered whole-brain connectivity patterns in the CID group compared with the normal control group. [file Image1.TIF]
